# COVID-19 mRNA Vaccination in Lactation: Assessment of Adverse Events and Vaccine Related Antibodies in Mother-Infant Dyads

**DOI:** 10.3389/fimmu.2021.777103

**Published:** 2021-11-03

**Authors:** Yarden Golan, Mary Prahl, Arianna G. Cassidy, Caryl Gay, Alan H. B. Wu, Unurzul Jigmeddagva, Christine Y. Lin, Veronica J. Gonzalez, Emilia Basilio, Megan A. Chidboy, Lakshmi Warrier, Sirirak Buarpung, Lin Li, Amy P. Murtha, Ifeyinwa V. Asiodu, Nadav Ahituv, Valerie J. Flaherman, Stephanie L. Gaw

**Affiliations:** ^1^Department of Bioengineering and Therapeutic Sciences, University of California, San Francisco, San Francisco, CA, United States; ^2^Institute for Human Genetics, University of California, San Francisco, San Francisco, CA, United States; ^3^Department of Pediatrics, University of California, San Francisco, San Francisco, CA, United States; ^4^Division of Pediatric Infectious Diseases and Global Health, University of California, San Francisco, San Francisco, CA, United States; ^5^Division of Maternal-Fetal Medicine, Department of Obstetrics, Gynecology, and Reproductive Sciences, University of California San Francisco, San Francisco, CA, United States; ^6^Department of Family Health Care Nursing, University of California, San Francisco, San Francisco, CA, United States; ^7^Department of Laboratory Medicine, University of California, San Francisco, San Francisco, CA, United States; ^8^Center for Reproductive Sciences, Department of Obstetrics, Gynecology, and Reproductive Sciences, University of California San Francisco, San Francisco, CA, United States; ^9^Department of Medicine, University of California, San Francisco, San Francisco, CA, United States

**Keywords:** COVID-19, SARS-CoV-2, lactation, antibodies, breastfeeding, human milk, mRNA vaccine, passive immunity

## Abstract

**Background:**

Data regarding symptoms in the lactating mother-infant dyad and their immune response to COVID-19 mRNA vaccination during lactation are needed to inform vaccination guidelines.

**Methods:**

From a prospective cohort of 50 lactating individuals who received mRNA-based vaccines for COVID-19 (mRNA-1273 and BNT162b2), blood and milk samples were collected prior to first vaccination dose, immediately prior to 2nd dose, and 4-10 weeks after 2nd dose. Symptoms in mother and infant were assessed by detailed questionnaires. Anti-SARS-CoV-2 antibody levels in blood and milk were measured by Pylon 3D automated immunoassay and ELISA. In addition, vaccine-related PEGylated proteins in milk were measured by ELISA. Blood samples were collected from a subset of infants whose mothers received the vaccine during lactation (4-15 weeks after mothers’ 2nd dose).

**Results:**

No severe maternal or infant adverse events were reported in this cohort. Two mothers and two infants were diagnosed with COVID-19 during the study period before achieving full immune response. PEGylated proteins were not found at significant levels in milk after vaccination. After vaccination, levels of anti-SARS-CoV-2 IgG and IgM significantly increased in maternal plasma and there was significant transfer of anti-SARS-CoV-2-Receptor Binding Domain (anti-RBD) IgA and IgG antibodies to milk. Milk IgA levels after the 2nd dose were negatively associated with infant age. Anti-SARS-CoV-2 IgG antibodies were not detected in the plasma of infants whose mothers were vaccinated during lactation.

**Conclusions:**

COVID-19 mRNA vaccines generate robust immune responses in plasma and milk of lactating individuals without severe adverse events reported.

## Introduction

An important benefit of human milk is the presence of IgA and IgG antibodies that provide passive immunity to the infant (1, 2). Anti-SARS-CoV-2 antibodies are present in milk from lactating women who were infected with SARS-CoV-2 (3, 4) or who received COVID-19 mRNA vaccines (5–14). Specifically, high titers of anti-SARS-CoV-2 IgG were reported after vaccination (6). In addition, IgG levels in milk were higher after vaccination compared to convalescent samples after SARS-CoV-2 infection (6, 14). The function of these antibodies in protection of infants against COVID-19 is not fully understood. In addition, it has recently been shown in a few studies that vaccines mRNA components are not present in milk samples after vaccination (15, 16), or were only detected in very low levels in some cases (14), providing reassurance that risks of exposure to the breastfed infant are minimal. Even though the infant receives passive immune protection from milk antibodies after vaccination, there is still significant hesitancy in the lactating population. Much of the concern is due to the lack of knowledge about the effect of mRNA-based vaccines on the nursing infant, as lactating mothers were excluded from initial clinical trials of mRNA vaccination (17). More studies that follow up on breastfeeding individuals and their infants after vaccination are needed to address concerns regarding the potential effects on infants, in order to prevent further delays in vaccination or early cessation of breastfeeding (18). In this study, we examined blood and milk samples from lactating mothers who received a COVID-19 mRNA vaccine, and their infants for the presence of anti- SARS-CoV2 antibodies and milk samples for the presence of PEGylated proteins which are part of the mRNA-based vaccines lipid nano-particles. In addition, we examine self-reported vaccine-related symptoms in order to address the gap of knowledge regarding vaccination efficacy and safety during lactation.

## Methods

### Study Approval and Study Population

The institutional review board of the University of California San Francisco approved the study. Written, informed consent was obtained from all study volunteers in the COVID-19 Vaccine in Pregnancy and Lactation (COVIPAL) cohort study from December 2020 to June 2021. Eligible participants were actively lactating, planning to receive any COVID-19 vaccine, and willing to donate blood and/or milk samples.

### Clinical Data Collection

Clinical data on vaccine side effects were collected through an online questionnaire that was sent to participants 21 days or more after each vaccine administration. Questionnaires were distributed using REDCap.

### Sample Collection

Maternal blood and milk samples were collected at three time points: 1) up to 1 day before the 1st dose (pre-vaccine); 2) on the day of and prior to administration of the 2nd dose (after 1st dose); and between 4-10 weeks after the 2nd dose (after 2nd dose). In some cases, additional milk samples were collected up to 31 days before the 1st dose, 24 hours after each dose, and weekly for up to 4 weeks after the 2nd dose. Infant blood was collected by heel stick by trained study staff at 5-15 weeks after 2nd maternal vaccination.

### Milk Processing

Fresh human milk samples were self-collected by participants into sterile containers at several time points before, during, and after vaccination. Milk samples were either collected immediately by the study staff or frozen by mothers in their home freezer as soon as possible after pumping. Samples were kept on ice during transport from home to the lab for processing. Milk was aliquoted and stored in -80°C until analyzed.

### Measurement of SARS-CoV-2 Specific IgM and IgG in Plasma Samples

Whole blood was collected into tubes containing EDTA. Plasma was isolated from whole blood by centrifugation and immediately cryopreserved at -80°C until analysis. Anti-SARS-CoV-2 plasma IgM and IgG antibodies were measured using the Pylon 3D automated immunoassay system (19) (ET Healthcare, Palo Alto, CA). In brief, quartz glass probes pre-coated with either affinity-purified goat anti-human IgM (IgM capture) or Protein G (IgG capture) were dipped into diluted plasma samples, washed, and then dipped into the assay reagent containing both biotinylated, recombinant spike protein receptor binding domain (RBD) and nucleocapsid protein (NP). After washing, the probes were incubated with Cy^®^5-streptavidin (Cy5-SA) polysaccharide conjugate reagent, allowing for cyclic amplification of the fluorescence signal. The background-corrected signal of SARS-CoV-2 specific antibodies was reported as relative fluorescent units (RFU). IgM and IgG measurements greater than 50 RFU were considered positive RFUs.

### Measurement of IgA and IgG by ELISA Assay in Milk

After thawing, milk fat was separated by cold centrifugation (10,000g for 10 min, 4°C). Milk supernatant samples were diluted 1:2 in sample diluent buffer and were plated in duplicate on a 96-well plate containing S1 spike protein RBD (Ray-Biotech, GA, USA, IEQ-CoVS1RBD-IgG-1 and IEQ-CoVS1RBD-IgA-1). For monomeric IgA assays, samples were also plated in duplicate on a second 96-well plate coated with human albumin to account for non-specific binding. OD values for albumin were subtracted from the OD values for RBD. Each plate contained seven wells of serial dilutions (1:3) of a positive control from an inactivated serum sample which contains SARS-COV-2 S1 RBD protein human IgA antibody (provided with the kit) and one blank negative control. The mean absorbance of each sample was captured on an ELISA plate reader at 450 nm. Background values (blank negative control) were subtracted from the albumin and RBD plates. Standard controls were used to create a standard curve and determine the level of anti-RBD IgA and IgG in unit/ml.

*Measurement of Polyethylene Glycol (PEGylated) proteins in human milk by ELISA*. Milk supernatant was diluted 1:8 with the provided sample buffer and analyzed by PEGylated protein ELISA kit (Enzo, Farmingdale, NY, USA). Seven wells of each plate were loaded with serial dilutions (1:2) of mRNA-1273 or BNT162b2 to generate the standard curve for each vaccine (**Figure S1A**). The PEGylated Protein ELISA kit is a competitive assay specific to the backbone of PEG. Samples and controls were loaded on plates pre-coated with monoclonal antibody to PEG which binds in a competitive manner the PEG or PEGylated protein in the sample, or the PEG covalently linked to biotin which is mixed and incubated together with the tested samples. Due to the competitive assay, the amount of signal (OD) is inversely proportional to the concentration of PEG in the sample (**Figure S1A**). To ensure the ability of the kit to detect the vaccine PEGylated components in milk samples, mRNA-1273 or BNT162b2 vaccines were separately inoculated into human milk samples at three different concentrations (33µl/ml, 3.3µl/ml and 0.33µl/ml) and were analyzed separately (**Figure S1B**). Prism 9 (v 9.1.2) was used to interpolate PEGylated proteins concentration in the samples based on OD values, using a sigmoidal, four parameters logistic curve. Standard curve for mRNA-1273 or BNT162b2 were used to analyze participant milk samples based on the vaccine received. Of note, the assay measures all types of PEGylated proteins (if present in the sample), and not only the vaccine PEGylated proteins.

### Statistics

All data analyses were conducted using Stata statistical software (v14, College Station, TX). Descriptive statistics included frequencies for categorical variables, and means, standard deviations, medians, and ranges for continuous variables. Group differences in categorical variables were analyzed using Fisher’s exact test, and group differences in continuous variables were analyzed using Mann-Whiney U tests. McNemar tests were used to evaluate differences in symptom frequencies after each vaccine dose. Spearman correlation was used to assess the magnitude of associations between continuous variables. Non-parametric tests were used to accommodate non-normal distributions and small group sizes.

## Results

### Participant Characteristics

During the study period, 50 participants answered all study questionnaires, provided blood and/or milk samples, had an infant up to 18 months old were included in this analysis. Two infants were diagnosed with COVID-19 during this study (**Table S1**, infant of participants 1 and 2). One mother reported that her infant had mild symptoms 1 week after the 2nd dose (not exclusively breastfed); this infant’s vaccinated mother had a negative test at the time of the infant’s positive PCR test. A second infant (exclusively breastfed) had positive plasma anti-SARS-CoV-2 IgG and IgA, despite the mother receiving the vaccine postpartum and reported no known prior COVID-19 infection. The mother’s plasma was subsequently found to be positive to antibodies against SARS-CoV-2 nucleocapsid protein, indicating a likely natural asymptomatic SARS-CoV-2 infection (further details in **Table S1**). Two mothers were positive for COVID-19 and are presented in **Table S1** (participants 2 and 3); they were excluded from further analysis of symptomatology. Cohort characteristics are presented in **Table 1**. Twenty-seven female participants [mean age 35.7 years (± 3.9)] received the BNT162b2 vaccine (Pfizer, 56%), and 21 received the mRNA-1237 (Moderna, 44%). The mean infant age at mother’s 1st dose was 5 months (± 3.9). All mothers continued to feed their infants with milk at the time of the 2nd vaccination, and all except one continued up to the time of follow up sample collection (4-10 weeks after 2nd dose). There were no significant differences in maternal or infant characteristics by vaccine manufacturer.

**Table 1:** Sample characteristics overall and by vaccine manufacturer.

Sample Characteristics	Full Cohort (n = 48, 100%)	BNT162b2 (n = 27, 56%)	mRNA-1237 (n = 21, 44%)
***Maternal characteristics***			
Maternal age, years			
Median (min, max)	35 (27, 46)	35 (30, 45)	35 (27, 46)
Race/ethnicity, % (n)			
Asian	31% (15)	30% (8)	33% (7)
Black or African American	2% (1)	4% (1)	0% (0)
White/Caucasian	59% (28)	55% (15)	62% (13)
Other (Middle Eastern)	2% (1)	0% (0)	5% (1)
More than 1 race/ethnicity (White+Latina/Asian/Middle Eastern)	6% (3)	11% (3)	0% (0)
Highest level of education completed			
Some college	2% (1)	0% (0)	5% (1)
College graduate	17% (8)	19% (5)	14% (3)
Advanced degree	81% (39)	81% (22)	81% (17)
Work in health care?			
Yes, providing direct patient care	58% (28)	52% (14)	67% (14)
Yes, but not in direct patient care	19% (9)	15% (4)	24% (5)
No	23% (11)	33% (9)	9% (2)
Pre-Pregnancy Body Mass Index			
Median (min, max)	23.4 (19.1, 37.5)	23.4 (19.1, 35.9)	22.8 (19.6, 37.5)
Number of children			
1	40% (19)	41% (11)	38% (8)
2	46% (22)	41% (11)	52% (11)
3	12% (6)	15% (4)	10% (2)
4	2% (1)	3% (1)	0% (0)
Duration of most recent pregnancy, weeks			
Median (min, max)	39.0 (33.9, 41.1)	39.1 (33.9, 41.0)	39.0 (37.4, 41.1)
***Infant characteristics***			
Infant age at maternal 1st dose, months			
Median (min, max)	4.7 (0.1, 17.2)	4.8 (0.2, 15.2)	4.6 (0.1, 17.2)
Sex, % (n)			
Male	60% (29)	67% (18)	52% (11)
Female	40% (19)	33% (9)	48% (10)
Exclusively breastfeeding (and no solids)			
Yes	23% (11)	22% (6)	24% (5)
No	77% (37)	78% (21)	76% (16)
***Days after vaccine that symptoms were assessed***			
Dose 1			
Mean (SD)	78.7 (31.8)	78.3 (35.4)	79.2 (27.4)
Median (min, max)	81 (18, 154]	78 (18, 154)	86 (26, 117)
Dose 2			
Mean (SD)	59.6 (25.1)	62.6 (28.3)	55.8 (20.2)
Median (min, max)	58.5 (28, 133)	57 (29, 133)	60 (28, 89)

None of the characteristics above differed significantly by vaccine manufacturer.

Standard deviation (SD), minimum (min), maximum (max).

### Post Vaccination Symptoms

Self-reported symptoms after each vaccine dose are presented in **Table 2**. Fever, chills, headache, joint pain, muscle aches or body aches, and fatigue or tiredness were reported by significantly more participants after the 2nd dose than after the 1st dose (**Table 2**). All 21 participants (100%) who received the mRNA-1237 vaccine reported injection site symptoms, while only 21 (78%) of 27 BNT-162b2 recipients reported injection site symptoms (p=0.02) (**Table 2**). Two mothers reported slightly less milk production in the first 24-72 hours after vaccine doses (**Table 2**). With respect to infant symptoms, 12% of mothers reported at least one symptom after the 1st maternal vaccine dose (primarily gastrointestinal symptoms and sleep changes), and none reported an infant symptom after the 2nd dose (**Table 3**). In summary, no severe adverse events (death, life threatening, hospitalization, disability) for mothers or nursing infants were reported in this cohort after vaccination, and reported symptoms resolved up to 72 hours after vaccination.

**Table 2:** Symptoms after each vaccine dose.

Symptoms	Full Cohort:			After 1st dose			After 2nd dose		
	1st dose	2nd dose	p-value^a^	BNT162b2	mRNA-1237	p-value^b^	BNT162b2	mRNA-1237	p-value^b^
	n = 48			n = 27	n = 21		n = 27	n = 21	
**Injection site symptoms, % (n)**									
Any injection site symptoms	88% (42)	88% (42)	>0.99	78% (21)	100% (21)	**0.02**	78% (21)	100% (21)	**.02**
Pain	88% (42)	85% (41)	0.71	78% (21)	100% (21)	**0.02**	78% (21)	95% (20)	0.12
Redness	4% (2)	10% (5)	0.08	0% (0)	10% (2)	0.19	4% (1)	19% (4)	0.15
Swelling	17% (8)	17% (8)	>0.99	7% (2)	29% (6)	0.12	11% (3)	24% (5)	0.27
Itching	4% (2)	4% (2)	>0.99	4% (1)	5% (1)	>.99	4% (1)	5% (1)	>.99
Rash around injection site	2% (1)	4% (2)	0.32	0% (0)	5% (1)	0.44	0% (0)	10% (2)	0.19
**Generalized symptoms, % (n)**									
Any general symptoms	48% (23)	92% (44)	**<0.001**	44% (12)	52% (11)	0.77	85% (23)	100% (21)	0.12
Fever	12% (6)	62% (30)	**<0.001**	19% (5)	5% (1)	0.21	52% (14)	76% (16)	0.13
Chills	8% (4)	48% (23)	**<0.001**	11% (3)	5% (1)	0.62	37% (10)	62% (13)	0.14
Headache	21% (10)	67% (32)	**<0.001**	11% (3)	33% (7)	0.08	56% (15)	81% (17)	0.07
Joint pain	8% (4)	31% (15)	**0.002**	7% (2)	10% (2)	>0.99	30% (8)	33% (7)	>0.99
Muscle/body aches	21% (10)	69% (33)	**<0.001**	30% (8)	10% (2)	0.15	59% (16)	81% (17)	0.13
Fatigue or tiredness	44% (21)	81% (39)	**<0.001**	41% (11)	48% (10)	0.77	67% (18)	100% (21)	**0.003**
Nausea	4% (2)	12% (6)	0.10	4% (1)	5% (1)	>0.99	7% (2)	19% (4)	0.38
Vomiting	0% (0)	0% (0)	—	0% (0)	0% (0)	—	0% (0)	0% (0)	—
Diarrhea	4% (2)	4% (2)	>0.99	4% (1)	5% (1)	>0.99	0% (0)	10% (2)	0.19
Abdominal pain	2% (1)	0% (0)	0.32	0% (0)	5% (1)	0.44	0% (0)	0% (0)	—
Rash not near injection site	0% (0)	0% (0)	—	0% (0)	0% (0)	—	0% (0)	0% (0)	—
Lump/swelling in breast (same side as injection)	0% (0)	2% (1)	0.32	0% (0)	0% (0)	—	4% (1)	0% (0)	>0.99
Lump/swelling in breast (opposite side as injection)	0% (0)	0% (0)	—	0% (0)	0% (0)	—	0% (0)	0% (0)	—
Mastitis	2% (1)	0% (0)	0.32	0% (0)	5% (1)	0.44	0% (0)	0% (0)	—
Decrease in milk supply	2% (1)	2% (1)	>0.99	0% (0)	5% (1)	0.44	0% (0)	5% (1)	0.44

a McNemar’s test.

b Fisher’s Exact test. Statistically significant values (p<0.05) are indicated in bold.

**Table 3:** Infant symptoms reported after maternal vaccination (write-in only).

INFANT SYMPTOMS	% (n)	Vaccine
After 1st vaccine dose		
None/no changes/blank	88% (42)	
* “My baby seemed a little tired.”*	2% (1)	BNT162b2
* “He started pooping a lot! And it was more sour smelling diarrhea like poops. I don’t know if there is any correlation”*	2% (1)	BNT162b2
* “It could have been a fluke, but both my infant and I slept through the night for the first time the night after I received the 1st dose of the vaccine.”*	2% (1)	BNT162b2
* “He had some diaper rash, but likely unrelated”*	2% (1)	BNT162b2
* “Rash on the face/worsening of baby acne”*	2% (1)	mRNA-1237
* “Disrupted sleep, waking at night when he usually doesn’t. More fussy than normal.”*	2% (1)	mRNA-1237
After 2nd vaccine dose		
None/no changes/blank	100% (48)	

### PEG Detection in Human Milk

**Figure 1:**
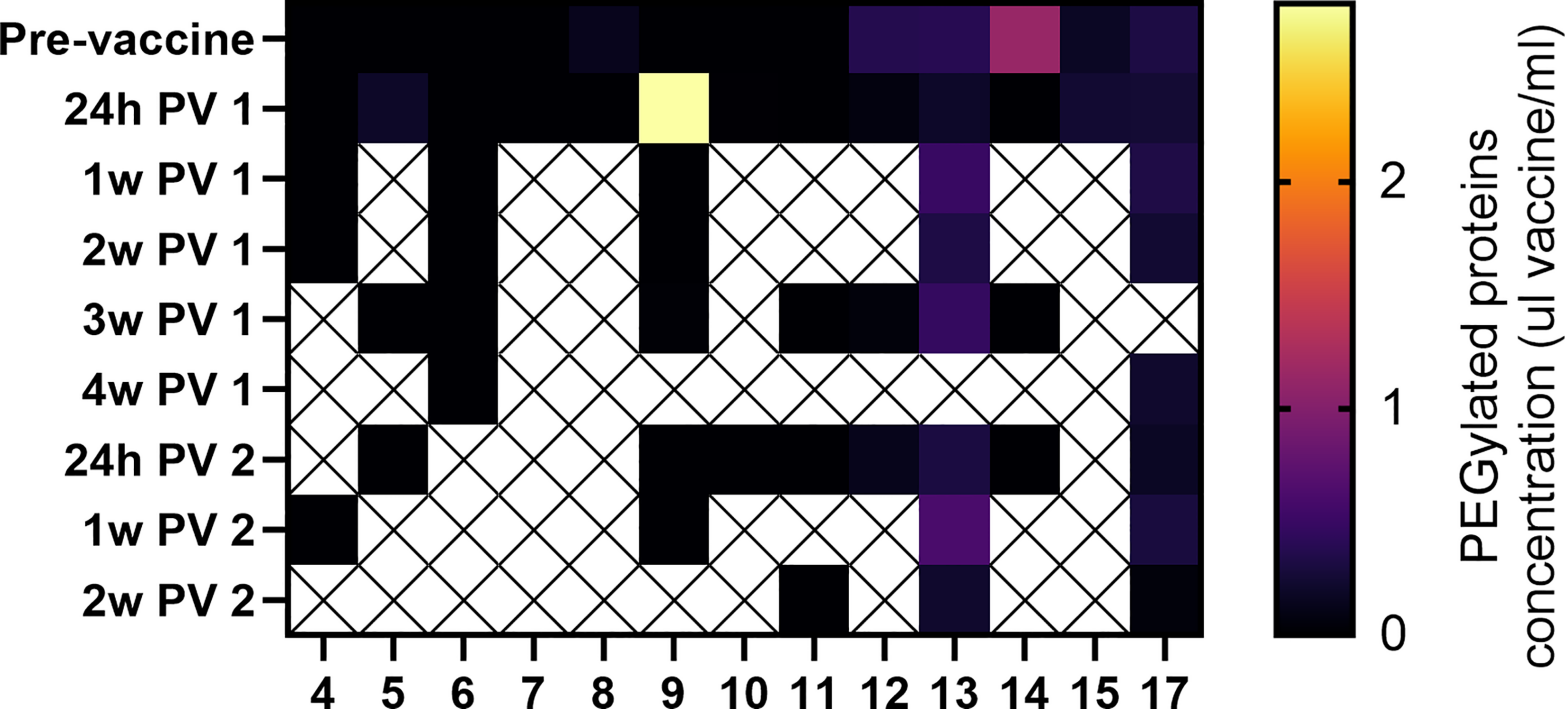
Detection of vaccine PEG in human milk samples. PEGylated protein concentration in each sample were interpolated based on vaccine standard curves (**Figure S1**). No significant differences were observed between samples collected at any of the post vaccine (PV) time points and the pre-vaccine samples (paired and unpaired two-tailed t-tests). Y axes represent time of sample collection, as hours (h) or weeks (w) Post vaccine 1 (PV 1), or Post vaccine 2 (PV 2).

Polyethylene glycol (PEG) is present in the lipid nanoparticles of the mRNA-based vaccines, and was reported to cause allergic reaction after vaccination in rare cases (20, 21). To address concerns about vaccine components passing to milk after vaccination, we performed ELISA assays to measure PEGylated proteins levels in milk after vaccination from 13 participants. PEGylated proteins were measured in milk samples collected before the vaccine, and at various time points post-vaccination (from 24 hours after 1st dose to 2 weeks after 2nd dose). Pre-vaccine PEGylated proteins concentration did not significantly differ from PEGylated proteins levels at any post-vaccine time point in either paired or unpaired comparisons (**Figure 1**).

### Anti-SARS-CoV-2 Antibody Levels in Blood and Milk Samples After Vaccination

**Figure 2:**
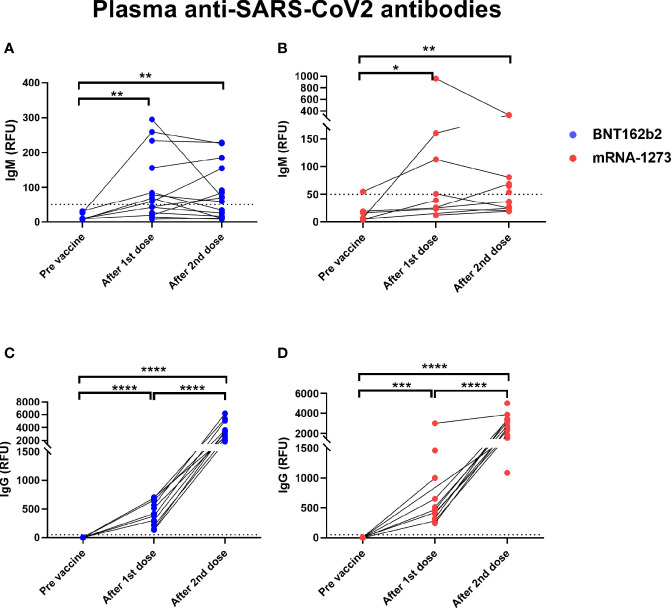
Elevated levels of plasma anti-SARS-CoV2 antibodies in COVID-19 mRNA vaccinated lactating individuals. Anti-SARS-CoV2 IgM levels in plasma of lactating individuals receiving BNT-162b2 (n=19) **(A)** and mRNA-1273 (n=13) **(B)** COVID-19 vaccines (RFU- relative fluorescent units, dashed line represents positive cut-off >50 RFU). Anti-SARS-CoV2 IgG levels in plasma of lactating individuals receiving BNT-162b2 **(C)** and mRNA-1273 **(D)** COVID-19 vaccines. After 1st dose samples were collected on the day of the second vaccine, and after 2nd dose samples were collect 4-10 weeks post 2nd dose. Asterisks represent p-values: *= p-value <0.05, **= p-value <0.01, ***= <0.001, ****= <0.0001 as determined by unpaired Mann-Whitney test.

We analyzed blood and milk samples from lactating individuals for anti-SARS-CoV-2 antibodies to measure immune response after vaccination. Maternal blood anti-SARS-CoV-2 IgM and IgG antibodies increased significantly after the 1st dose **(****Figure 2****)**. Anti-SARS-CoV-2 IgM levels were not significantly higher 4-10 weeks after the 2nd dose compared to samples collected after dose 1 (on the day of the 2nd dose) (**Figures 2A, B****)**. In contrast, anti-SARS-CoV-2 IgG levels increased significantly after the 2nd dose (P value <0.0001) when compared to samples collected immediately prior to the 2nd dose (**Figures 2C, D****)**. There was no significant difference in blood antibody levels between participants who received the mRNA-1237 compared to the BNT-162b2 vaccine after dose 2 (determined by unpaired Mann-Whitney test).

**Figure 3:**
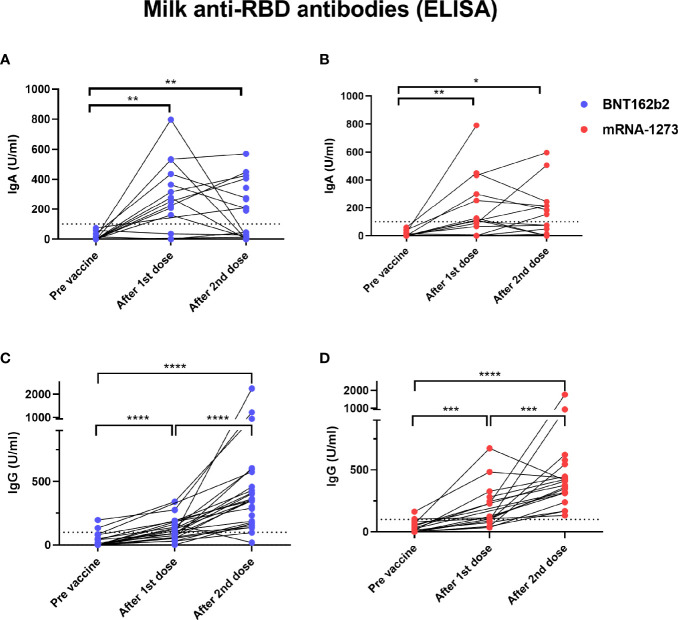
Elevated levels of milk anti-SARS-CoV2 IgA antibodies in COVID-19 mRNA vaccinated lactating individuals. Milk samples from individuals receiving BNT-162b2 (n=27) **(A)** and mRNA-1273 (n=21) **(B)** COVID-19 vaccines were analyzed for anti-SARS-CoV2 IgA antibodies using ELISA at various time points as indicated on the X axis. After 1st dose samples were collected on the day of the second vaccine, and after 2nd dose samples were collect 4-10 weeks post 2nd dose. Milk anti-SARS-CoV2 IgG levels were measured using ELISA in milk samples from individuals receiving BNT-162b2 (n=27) **(C)** or mRNA-1273 (n=21) **(D)**. Asterisks represent p-values: *= p-value <0.05, **= p-value <0.01, ***= <0.001, ****= <0.0001 as determined by unpaired Mann-Whitney test. Dashed line represents positive cut-off >100 U/ml.

We found significantly higher levels of IgA antibodies specific to SARS-CoV-2 RBD protein in human milk samples collected after the 1st dose of both BNT-162b2 and mRNA-1237 vaccines (**Figures 3A, B****)**. There was no significant increase in milk anti-RBD IgA after the 2nd vaccination as compared to after dose 1 (**Figures 3A, B**). Twelve individuals (25%, BNT-162b2 n=7; mRNA-1237 n=5) did not have detectable levels of anti-RBD IgA after either the 1st or 2nd dose (infants age at 1st dose range 1-11 months). Milk anti-RBD IgG levels increased after the 1st dose of vaccine and increased further after the 2nd dose (**Figures 3C, D****)**. There were no significant differences in milk anti-RBD IgG levels between women who received BNT-162b2 (**Figure 3C****)** and mRNA-1237 (**Figure 3D****)**. These findings suggest that mRNA vaccine results in a robust immune response leading to increased anti SARS-CoV-2 antibody levels in blood, but also in milk during lactation.

### Correlations Between Antibody Levels, Participant Characteristics, and Symptoms

**Figure 4:**
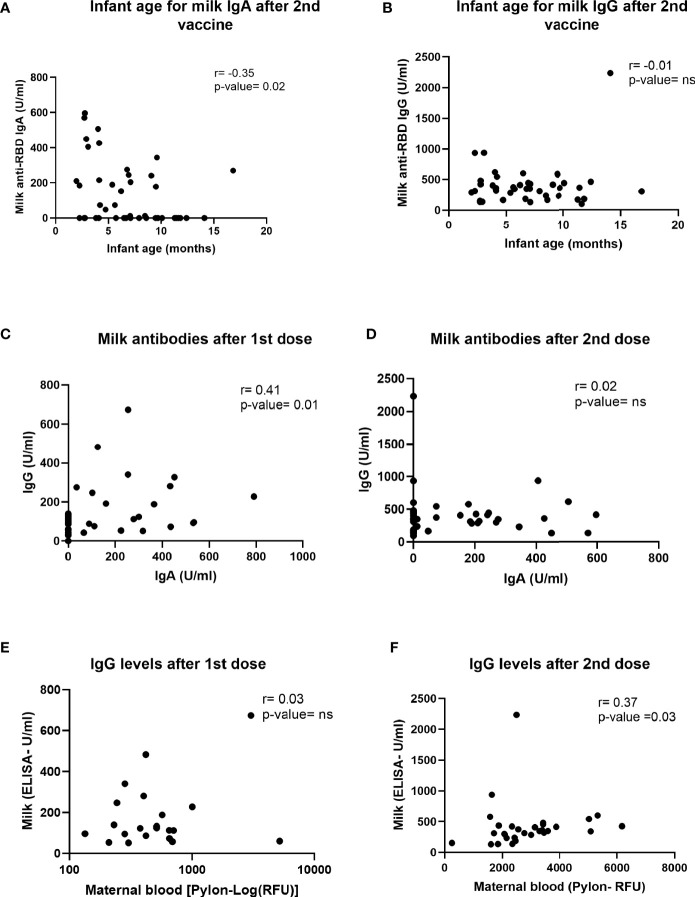
Correlations between milk antibodies, blood antibodies and infant age. Two-tailed Spearman correlation was used to correlate milk IgA **(A)** and IgG **(B)** levels (Y axis) and infant age (X axis) 4-10 weeks after the 2nd dose administration (n=30). In addition, two-tailed Spearman correlation was used to correlate milk IgG (Y axis) and milk IgA levels (X axis) on the day of 2nd dose administration **(C)**, 21-28 days after 1st dose (n=35) and 4-10 weeks after 2nd dose **(D)**. We also tested correlation between milk (Y axis) and maternal plasma (X axis) IgG levels at day of 2nd dose **(E)** and 4-10 weeks after the 2nd dose administration **(F)** (n=30). Semi-partial correlations were used to assess relationships between variables while controlling for the effects of other relevant variables.

To better understand the differences in antibody responses between individuals in our cohort, we performed multiple correlation tests to determine whether IgG and IgA antibodies levels correlated with timing of sample collection after vaccination (range 4-10 weeks after 2nd dose), infant age at time of vaccination, or maternal BMI (**Table S2****)**. Milk IgA (but not IgG) levels measured after the second dose declined significantly as the infant age at time of vaccination increased **(****Figures 4A, B****)**. There was no significant correlation between IgG and IgA levels and either the length of time after 2nd dose or maternal BMI (**Tables S2, S3**). The levels of IgG and IgA antibodies induced in milk were significantly correlated after 1st dose (**Figure 4C****)**, but not after the 2nd dose (**Figure 4D**). There was no correlation between the anti-SARS-CoV-2 IgG levels in blood and milk after 1st dose (**Figure 4E**), but there was a positive correlation between levels at 4-10 weeks after 2nd dose (**Figure 4F**).

### Plasma Levels of Anti-SARS-CoV-2 IgG Are Not Detectable in Infants After Maternal Vaccination During Lactation

**Figure 5:**
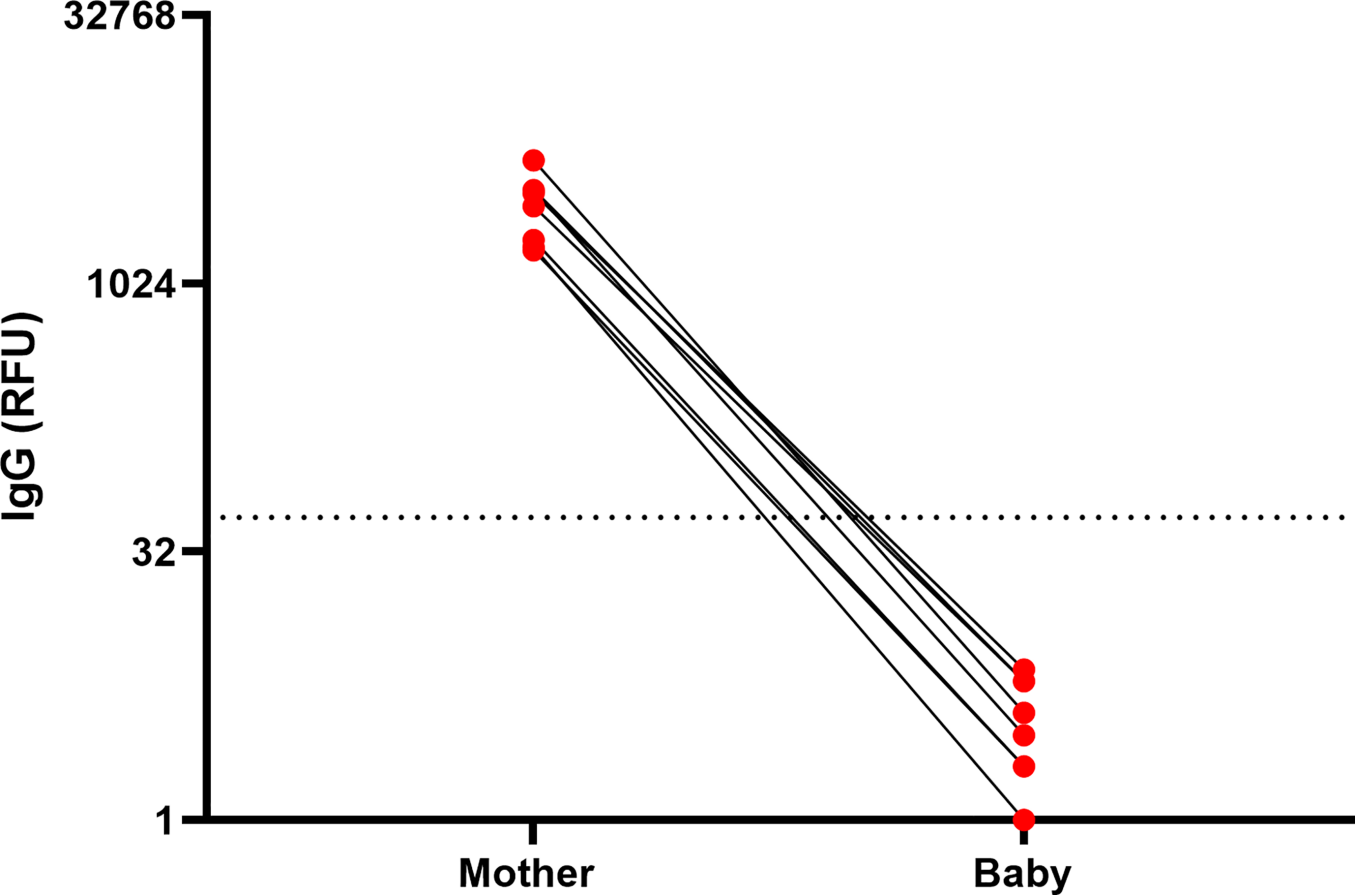
Infants anti-SARS-CoV2 IgG levels after maternal vaccination during lactation. IgG levels were measured in blood samples of infants and mothers 61 days to 1 year postpartum, 61-129 days after 1st maternal vaccine administration. Maternal and infant samples were collected in the same week (except in one case in which the maternal sample was collected 18 days prior to the infant sample). (RFU- relative fluorescent units, dashed line represents positive cut-off >50 RFU). Sample characteristics and individual antibodies levels are presented in **Table S4**.

Although maternal IgG antibodies have been shown in multiple studies to transfer to the infant *in utero*, existing data suggests that milk-derived antibodies are not transferred to the infant blood circulation during breastfeeding (22, 23). To investigate whether maternal vaccination during lactation triggers infant immune responses, we analyzed infant blood samples from a subset of infants in our cohort (n=8). Blood samples were collected from these 8 infants (4 male, 4 female) at 68 days to 1 year of age (**Table S4**). Plasma was tested for the presence of anti-SARS-CoV-2 IgG and IgM and anti-RBD IgA. We evaluated infant blood samples collected at time frame of 4-10 weeks after 2nd dose as this time point corresponded to high anti-SARS-CoV-2 IgG levels in mothers’ blood and milk (**Figures 2**, **3**). No antibodies were detected in the blood of nursing infants born to mothers who were vaccinated postpartum (**Figure 5**), despite high IgG levels in maternal blood and milk. In contrast, infants born to mothers who received both doses of vaccine during pregnancy had detectable plasma anti-SARS-CoV-2 IgG levels at birth (24) and at follow-up (data not showed). None of the follow-up infant blood samples had detectable levels of anti-RBD IgA antibodies. These results demonstrate that vaccination during lactation induces anti-SARS-CoV-2 antibodies in human milk but does not lead to detectable immunity in the infant, and does not provide additional transfer (or production) of anti-SARS-CoV-2 antibodies to the infant blood, in contrast to vaccination during pregnancy. Furthermore, maternal vaccination does not appear to stimulate an immune response in lactating infants, as expected.

## Conclusion

Our study provides a detailed report on patient symptoms and antibody responses of the COVID-19 mRNA vaccines in lactating mothers. We found that the rates of reported symptoms were similar to the CDC report from the V-Safe registry (25) but higher than described in the clinical trials (26, 27), although we do not have a non-lactating comparison group. Comparing the mRNA vaccines made by current manufacturers, we found that lactating individuals may experience more vaccine-related side effects after mRNA-1237 compared to BNT-162b2 vaccine. These findings were in line with a report from another survey-based cohort study (28). Although in some studies, mRNA-1237 vaccine was shown to induce higher Spike and RBD-specific IgA titters in blood (8), in our cohort we found no significant differences in immune response between those vaccines.

Importantly, no severe side effects were reported in the infants of mothers vaccinated during breastfeeding. The reported symptoms (primarily gastrointestinal symptoms, rash, and sleep changes) were also reported in a larger cohort of vaccinated lactation mothers in a relatively similar low frequencies (28). However, both our study and the study by McLaurin-Jiang et al, are missing a non-vaccinated control group. The reported symptoms are common in lactating infants and might not be directly related to vaccine administration but to viral infection of other factors. For example, a mother in our study (**Table S1****)**, mother 2 and her baby were diagnosed with COVID-19 (based on serologic testing), and she reported that the infant was “Less active. Feverish” after the 2nd dose, but she didn’t report the SARS-CoV-2 infection. Of note, we were able to confirm COVID-19 infection using anti-nucleocapsid antibody assay in this study; however, in other survey-based studies cases of infection cannot be ruled out, which may confound infant symptomatology reporting and assessments of associations with vaccination, rather than other mild viral infections. Further studies that compare symptoms in infants of vaccinated and non-vaccinated women are needed.

Our study found no significant increase in milk PEGylated protein concentrations at various time points after vaccine administration in a subset of samples analyzed in our cohort. We did observe one sample with higher concentration of PEGylated proteins 24 hours after 1st dose, compared to pre-vaccine sample (**Figure 1**, patient 9). This sample had PEGylated protein levels equivalent to 2.8µl/ml vaccine. However, we cannot confirm that the increased PEG in this single sample was from the COVID-19 vaccine, as PEG exposure may also be from other sources, such laxatives or ibuprofen. There was no increase in protein PEGylation concentration after 2nd dose in the same individual, and no unusual symptoms were reported in either the mother or her infant. These results demonstrate in a small cohort, that there is no significant increase in milk PEG levels after the first or second vaccination. Larger studies are needed to increase our understanding of the presence of PEG in human milk, and the biological relevance of these components after ingestion by the infant. Although expert consensus states there is minimal or no potential risk for the infant from maternal COVID-19 vaccination (29, 30), the minor symptoms that were reported (sleep changes and gastrointestinal symptoms) could be further investigated in future studies to determine if they are related to vaccination. Our findings also suggest that administration of maternal mRNA-based vaccine during lactation did not lead to a detectable immune response in the infant blood. These results further suggest that maternal vaccination during lactation cannot trigger infant immune responses to a degree that generates infant immunity.

We also demonstrate that COVID-19 mRNA vaccination induces significant increases in anti-SARS-CoV-2 IgM and IgG levels in lactating mothers’ blood. Consistent with previous studies that showed IgM levels plateaued 28 days after COVID-19 infection (31), our results also demonstrated that 2nd dose did not induce significantly higher levels of IgM than was observed after 1st dose. In contrast, maternal blood IgG levels increased by 6-fold after the 2nd dose (compare to the levels after the 1st dose), highlighting the importance of the 2nd dose to boost the antibody response (27). We also observed a similar pattern of increase in anti-RBD IgG levels in milk after the 2nd dose and positive correlation of blood and milk IgG levels. These findings stand in line with previous publications (32, 33) and strengthen our knowledge about the transport of milk IgG antibodies from the blood to the milk (34). In contrast, milk anti-RBD IgA levels measured 4-10 weeks after 2nd dose, were not significantly higher compared to their levels after the 1st dose. Spike-SIgA as well as IgA S2 titters in milk were previously reported to remain unaffected by 2nd vaccine dose (8) or to reach peak levels one week after the 2nd dose (5).

Twenty-five percent of women in our cohort had no detectable levels of anti-RBD IgA in their milk after vaccination. Similar findings were reported in other studies (5, 6), suggesting that production and transfer efficiency vary between individuals. Our analysis showed a weak but significant negative correlation between infant age and milk anti-RBD IgA levels, which might explain some of the variation in milk IgA levels observed between different individuals. Ten out of the 12 participants who had no detectable anti-RBD IgA, had infants older than 5.5 months at the time of sample collection. These findings are different from other publications that showed positive correlation of milk IgA levels (measured 2 weeks after 2nd dose) and baby age (32) or another study that didn’t show correlation between these antibodies titters in milk and infant age (10 days after 2nd dose) (35). In our study IgA levels in milk were measured 4-10 weeks post 2nd dose and based on other publications we expect that milk IgA levels will be relatively lower at this stage in compared to 10-14 days after 2nd dose. These differences in the timing of measurements might explain the differences in our findings, compared to other studies (32, 35). Although we did not measure maternal blood IgA, other studies have shown that blood and milk IgA levels correlate when measured 7-10 days after the 2nd dose (13, 36). The relationship between infant age, breastfeeding exclusivity, milk IgA antibodies, and optimal timing of vaccination during lactation remains to be studied in detail.

Due to the lack of data about vaccination during pregnancy, many pregnant individuals were initially denied access to, declined, or were recommended to delay vaccination until after pregnancy. As such, many mothers have waited until after delivery to receive the vaccine. Although mothers vaccinated during lactation transferred antibodies to their infant through milk, which is an important component of mucosal immunity for the baby, there was no passive transfer of antibodies to the infant bloodstream (**Figure 5**), as occurs if the mother is vaccinated during pregnancy (8). Correlates of infant immune protection to COVID-19 are not yet well understood, however passive *in utero* transfer of IgG to the infant is important in the prevention of a number of infections including pertussis and influenza (37–39). Passively-transferred milk-derived IgA and IgG likely provide partial mucosal immune protection in infants, as breastfeeding is associated with lower risk of infections associated with mucosal defense, especially against respiratory infections (40–42). Two nursing infants in our cohort were infected with COVID-19 during the study (one a week post maternal 2nd dose, and the second one between 1st and 2nd maternal vaccine), indicating that at the time before full immune response is achieved in the vaccinated mother, typically 2-3 weeks after the 2nd dose milk antibodies cannot fully protect against SARS-CoV-2 infection (**Figure 2D**) and especially if the infant is not exclusively breastfed. Further studies are needed to determine the degree of protection conferred by IgA and IgG anti-SARS-CoV-2 antibodies that are present in milk. In addition, studies evaluating the additive benefit of both transplacentally-derived maternal IgG, as well as milk-derived IgA and IgG are needed to determine protection against COVID-19 in early infancy. Our findings underscore the importance of determining the optimal timing of vaccine administration to confer maximal protection against COVID-19 in infancy.

Strengths of our study include the prospective design and comprehensive symptom reporting by the vaccinated participants. We also report on longitudinal follow-up of infant immune responses, which has not been previously described. Furthermore, we included both BNT162b2 and mRNA-1273 vaccines and compared responses between the two vaccine manufacturers. Limitations include the small sample size, and that not all samples were able to be collected from all infant participants.

In summary, our study reports that no severe adverse events were noted in lactating individuals or their breastfeeding infants after COVID-19 mRNA vaccination. We demonstrated that human milk confers passive immunity to the infants, primarily through mucosal immunity in the gastrointestinal tract provided by IgA and IgG in milk. These results are important evidence to aid in counseling lactating individuals on the safety and efficacy of the COVID-19 mRNA vaccines, and the potential benefits to both the mother and infant.

## Data Availability Statement

The raw data supporting the conclusions of this article will be made available by the authors, without undue reservation.

## Ethics Statement

The studies involving human participants were reviewed and approved by The institutional review board of the University of California San Francisco. The patients/participants provided their written informed consent to participate in this study.

## Author Contributions

YG, MP, VF, IA, and SG designed the study. AC and CL recruited participants. YG, MP, AC, UJ, AW, CL, VG, MC, LW, SB, LL, and EB conducted experiments and acquired data. YG, MP, CG, and SG analyzed data. YG, MP, AC, NA, and SG wrote the manuscript. MP, AM, and SG provided funding. All authors assisted with editing the manuscript. NA, VF, and SG supervised the study. All authors contributed to the article and approved the submitted version.

## Funding

These studies were supported by the Marino Family Foundation (to MP), the National Institutes of Health (NIAID K23AI127886 to M.P. and NIAID K08AI141728 to SG), the Krzyzewski Family (to AM and SG), the Weizmann Institute of Science -National Postdoctoral Award Program for Advancing Women in Science (to YG), the International Society for Research In Human Milk and Lactation (ISRHML) Trainee Bridge Fund (to YG), and of the Human Frontier Science Program (to YG).

## Conflict of Interest

The authors declare that the research was conducted in the absence of any commercial or financial relationships that could be construed as a potential conflict of interest.

## Publisher’s Note

All claims expressed in this article are solely those of the authors and do not necessarily represent those of their affiliated organizations, or those of the publisher, the editors and the reviewers. Any product that may be evaluated in this article, or claim that may be made by its manufacturer, is not guaranteed or endorsed by the publisher.

## References

[1] Labayo HKM Pajuelo MJ Tohma K Ford-Siltz LA Gilman RH Cabrera L. Norovirus-Specific Immunoglobulin a in Breast Milk for Protection Against Norovirus-Associated Diarrhea Among Infants. EClinicalMedicine (2020) 27:100561. doi: 10.1016/j.eclinm.2020.100561 33043286 PMC7536734

[2] Sadeharju K Knip M Virtanen SM Savilahti E Tauriainen S Koskela P. Maternal Antibodies in Breast Milk Protect the Child From Enterovirus Infections. Pediatrics (2007) 119:941–6. doi: 10.1542/peds.2006-0780 17473095

[3] Fox A Marino J Amanat F Krammer F Hahn-Holbrook J Zolla-Pazner S. Robust and Specific Secretory Iga Against SARS-CoV-2 Detected in Human Milk. iScience (2020) 23(11):101735. doi: 10.1016/j.isci.2020.101735 33134887 PMC7586930

[4] Demers-Mathieu V Dung M Mathijssen GB Sela DA Seppo A Järvinen KM. Difference in Levels of SARS-CoV-2 S1 and S2 Subunits- and Nucleocapsid Protein-Reactive Sigm/Igm, Igg and Siga/Iga Antibodies in Human Milk. J Perinatol (2020) 41(4):850–59. doi: 10.1038/s41372-020-00805-w PMC7461757 32873904

[5] Perl SH Uzan-Yulzari A Klainer H Asiskovich L Youngster M Rinott E. SARS-CoV-2–Specific Antibodies in Breast Milk After COVID-19 Vaccination of Breastfeeding Women. JAMA (2021) 325(19):2013–4. doi: 10.1001/jama.2021.5782 PMC8042567 33843975

[6] Fox A Norris C Amanat F Zolla-Pazner S Powell RL. The Vaccine-Elicited Immunoglobulin Profile in Milk After COVID-19 mRNA-Based Vaccination is Igg-Dominant and Lacks Secretory Antibodies. medRxiv (2021). doi: 10.1101/2021.03.22.21253831. 2021.03.22.21253831.

[7] Kelly JC Carter EB Raghuraman N Nolan LS Gong Q Lewis AN. Anti-SARS-CoV-2 Antibodies Induced in Breast Milk After Pfizer-Biontech/BNT162b2 Vaccination. Am J Obstet Gynecol (2021) 225(1):101–3. doi: 10.1016/j.ajog.2021.03.031 PMC8062573 33798480

[8] Gray KJ Bordt EA Atyeo C Deriso E Akinwunmi B Young N. COVID-19 Vaccine Response in Pregnant and Lactating Women: A Cohort Study. Am J Obstet Gynecol (2021) 225(3):303.E1–17. doi: 10.1016/J.AJOG.2021.03.023. 2021.03.07.21253094. PMC7997025 33775692

[9] Juncker HG Mulleners SJ Gils MJv de Groot CJM Pajkrt D Korosi A. The Levels of SARS-CoV-2 Specific Antibodies in Human Milk Following Vaccination. J Hum Lact (2021) 37:477–84. doi: 10.1177/08903344211027112 34176363

[10] Guida M Terracciano D Cennamo M Aiello F Civita E Esposito G. COVID-19 Vaccine mRNAbnt162b2 Elicits Human Antibody Response in Milk of Breastfeeding Women. Vaccines (2021) 9:785. doi: 10.3390/VACCINES9070785 34358201 PMC8310008

[11] Charepe N Gonçalves J Juliano AM Lopes DG Canhão H Soares H. COVID-19 mRNA Vaccine and Antibody Response in Lactating Women: A Prospective Cohort Study. BMC Pregnancy Childbirth (2021) 21:1–9. doi: 10.1186/S12884-021-04051-6. 211. 34535094 PMC8447894

[12] Baird JK Jensen SM Urba WJ Fox BA Baird JR. SARS-CoV-2 Antibodies Detected in Mother’s Milk Post-Vaccination. J Hum Lact (2021) 37:492–8. doi: 10.1177/08903344211030168 PMC8685565 34297643

[13] Jakuszko K Kościelska-Kasprzak K Żabińska M Bartoszek D Poznański P Rukasz D. Immune Response to Vaccination Against COVID-19 in Breastfeeding Health Workers. Vaccines (2021) 9:663. doi: 10.3390/VACCINES9060663 34204501 PMC8235492

[14] Low JM Gu Y Ng MSF Amin Z Lee LY Ng YPM. Codominant Igg and Iga Expression With Minimal Vaccine mRNA in Milk of BNT162b2 Vaccinees. NPJ Vaccines (2021) 6:1–8. doi: 10.1038/s41541-021-00370-z. 61. 34413319 PMC8376902

[15] Mattar CN Koh W Seow Y Hoon S Venkatesh A Dashraath P. Title Page Addressing Anti-Syncytin Antibody Levels, and Fertility and Breastfeeding Concerns, Following BNT162B2 COVID-19 mRNA Vaccination. medRxiv (2021) 2021. doi: 10.1101/2021.05.23.21257686. 05.23.21257686.

[16] Golan Y Prahl M Cassidy A Lin CY Ahituv N Flaherman VJ. Evaluation of Messenger RNA From COVID-19 BTN162b2 and mRNA-1273 Vaccines in Human Milk. JAMA Pediatr (2021) 175(10):1069–71. doi: 10.1001/jamapediatrics.2021.1929 PMC8261686 34228115

[17] Krause PR Gruber MF. Emergency Use Authorization of Covid Vaccines — Safety and Efficacy Follow-Up Considerations. N Engl J Med (2020) 383:e107. doi: 10.1056/nejmp2031373 33064383

[18] Hall S. COVID Vaccines and Breastfeeding: What the Data Say. Nature (2021) 594:492–4. doi: 10.1038/D41586-021-01680-X 34163051

[19] Lynch KL Whitman JD Lacanienta NP Beckerdite EW Kastner SA Shy BR. Magnitude and Kinetics of Anti-Severe Acute Respiratory Syndrome Coronavirus 2 Antibody Responses and Their Relationship to Disease Severity. Clin Infect Dis (2021) 72:301–8. doi: 10.1093/cid/ciaa979 PMC7454426 33501951

[20] Sellaturay P Nasser S Ewan P. Polyethylene Glycol–Induced Systemic Allergic Reactions (Anaphylaxis). J Allergy Clin Immunol Pract (2021) 9:670–5. doi: 10.1016/J.JAIP.2020.09.029 33011299

[21] Garvey LH Nasser S. Anaphylaxis to the First COVID-19 Vaccine: Is Polyethylene Glycol (PEG) the Culprit? BJA Br J Anaesth (2021) 126:e106. doi: 10.1016/J.BJA.2020.12.020 33386124 PMC7834677

[22] Albrecht M Arck PC. Vertically Transferred Immunity in Neonates: Mothers, Mechanisms and Mediators. Front Immunol (2020) 11:555. doi: 10.3389/FIMMU.2020.00555 32296443 PMC7136470

[23] de Perre PV. Transfer of Antibody *via* Mother’s Milk. Vaccine (2003) 21:3374–6. doi: 10.1016/S0264-410X(03)00336-0 12850343

[24] Beharier O Plitman Mayo R Raz T Nahum Sacks K Schreiber L Suissa-Cohen Y. Efficient Maternal to Neonatal Transfer of Antibodies Against SARS-CoV-2 and BNT162b2 mRNA COVID-19 Vaccine. J Clin Invest (2021) 131(13):e150319. doi: 10.1172/JCI150319 PMC8245182 34014840

[25] Shimabukuro TT Kim SY Myers TR Moro PL Oduyebo T Panagiotakopoulos L. Preliminary Findings of mRNA Covid-19 Vaccine Safety in Pregnant Persons. N Engl J Med (2021) 384:2273–82. doi: 10.1056/nejmoa2104983 PMC8117969 33882218

[26] Baden LR El Sahly HM Essink B Kotloff K Frey S Novak R. Efficacy and Safety of the mRNA-1273 SARS-CoV-2 Vaccine. N Engl J Med (2020) 384:403–16. doi: 10.1056/NEJMoa2035389 PMC7787219 33378609

[27] Polack FP Thomas SJ Kitchin N Absalon J Gurtman A Lockhart S. Safety and Efficacy of the BNT162b2 mRNA Covid-19 Vaccine. N Engl J Med (2020) 383:2603–15. doi: 10.1056/NEJMoa2034577 PMC7745181 33301246

[28] McLaurin-Jiang S Garner CD Krutsch K Hale TW. Maternal and Child Symptoms Following COVID-19 Vaccination Among Breastfeeding Mothers. Breastfeed Med (2021) 16:702–9. doi: 10.1089/BFM.2021.0079 34171971

[29] Stafford IA Parchem JG Sibai BM. The COVID-19 Vaccine in Pregnancy: Risks Benefits and Recommendations. Am J Obstet Gynecol (2021) 224(5):484–95. doi: 10.1016/j.ajog.2021.01.022 PMC7847190 33529575

[30] Craig AM Hughes BL Swamy GK. Coronavirus Disease 2019 Vaccines in Pregnancy. Am J Obstet Gynecol MFM (2021) 3:100295. doi: 10.1016/j.ajogmf.2020.100295 33516986 PMC7832570

[31] Liu X Wang J Xu X Liao G Chen Y Hu CH. Patterns of Igg and Igm Antibody Response in COVID-19 Patients. Emerg Microbes Infect (2020) 9:1269–74. doi: 10.1080/22221751.2020.1773324 PMC7448841 32515684

[32] Ramírez DSR Pérez MML Pérez MC Hernández MIS Pulido SM Villacampa LP. SARS-CoV-2 Antibodies in Breast Milk After Vaccination. Pediatrics (2021) 148(5):35–43. doi: 10.1542/PEDS.2021-052286 34408089

[33] Esteve-Palau E Gonzalez-Cuevas A Guerrero ME Garcia-Terol C Alvarez MC Casadevall D. Quantification of Specific Antibodies Against SARS-Cov-2 in Breast Milk of Lactating Women Vaccinated With an mRNA Vaccine. JAMA Netw Open (2021) 4(8):e2120575. doi: 10.1001/JAMANETWORKOPEN.2021.20575 34379127 PMC8358734

[34] Atyeo C Alter G. The Multifaceted Roles of Breast Milk Antibodies. Cell (2021) 184:1486–99. doi: 10.1016/j.cell.2021.02.031 33740451

[35] Gonçalves J Juliano AM Charepe N Alenquer M Athayde D Ferreira F. Non-Neutralizing Secretory Iga and T Cells Targeting SARS-Cov-2 Spike Protein Are Transferred to the Breastmilk Upon BNT162b2 Vaccination. medRxiv (2021) 2021. doi: 10.1101/2021.05.03.21256416. 05.03.21256416. PMC8636305 34873588

[36] Valcarce V Stafford LS Neu J Cacho N Parker L Mueller M. Detection of SARS-Cov-2-Specific Iga in the Human Milk of COVID-19 Vaccinated Lactating Health Care Workers. Breastfeed Med (2021). doi: 10.1089/BFM.2021.0122 34427487

[37] Dabrera G Amirthalingam G Andrews N Campbell H Ribeiro S Kara E. A Case-Control Study to Estimate the Effectiveness of Maternal Pertussis Vaccination in Protecting Newborn Infants in England and Wales, 2012-2013. Clin Infect Dis (2015) 60:333–7. doi: 10.1093/cid/ciu821 25332078

[38] Amirthalingam G Andrews N Campbell H Ribeiro S Kara E Donegan K. Effectiveness of Maternal Pertussis Vaccination in England: An Observational Study. Lancet (2014) 384:1521–8. doi: 10.1016/S0140-6736(14)60686-3 25037990

[39] Puck JM Glezen WP Frank AL Six HR. Protection of Infants From Infection With Influenza a Virus by Transplacentally Acquired Antibody. J Infect Dis (1980) 142:844–9. doi: 10.1093/infdis/142.6.844 7462695

[40] Wright AL Holberg CJ Martinez FD Morgan WJ Taussig LM. Breast Feeding and Lower Respiratory Tract Illness in the First Year of Life. Group Health Medical Associates. BMJ (1989) 299:946–9. doi: 10.1136/bmj.299.6705.946 PMC1837795 2508946

[41] Tarrant M Kwok M Lam T Epidemiology G--. Breast-Feeding and Childhood Hospitalizations for Infections. JSTOR (2010) 21(6):847–54. doi: 10.1097/EDE.0b013e3181f55803 20864890

[42] Oddy WH Sly PD De Klerk NH Landau LI Kendall GE Holt PG. Breast Feeding and Respiratory Morbidity in Infancy: A Birth Cohort Study. Arch Dis Child (2003) 88:224–8. doi: 10.1136/adc.88.3.224 PMC1719488 12598384

