# Supplementary material for: COVID-19 mRNA Vaccination in Lactation: Assessment of Adverse Events and Vaccine Related Antibodies in Mother-Infant Dyads
Source: Front Immunol. 2021 Nov 3;12:777103. doi: 10.3389/fimmu.2021.777103 (PMC8595828; doi:10.3389/fimmu.2021.777103)
Supplement: Supplementary file 1 [file DataSheet_1.docx]

**Supplementary data:**


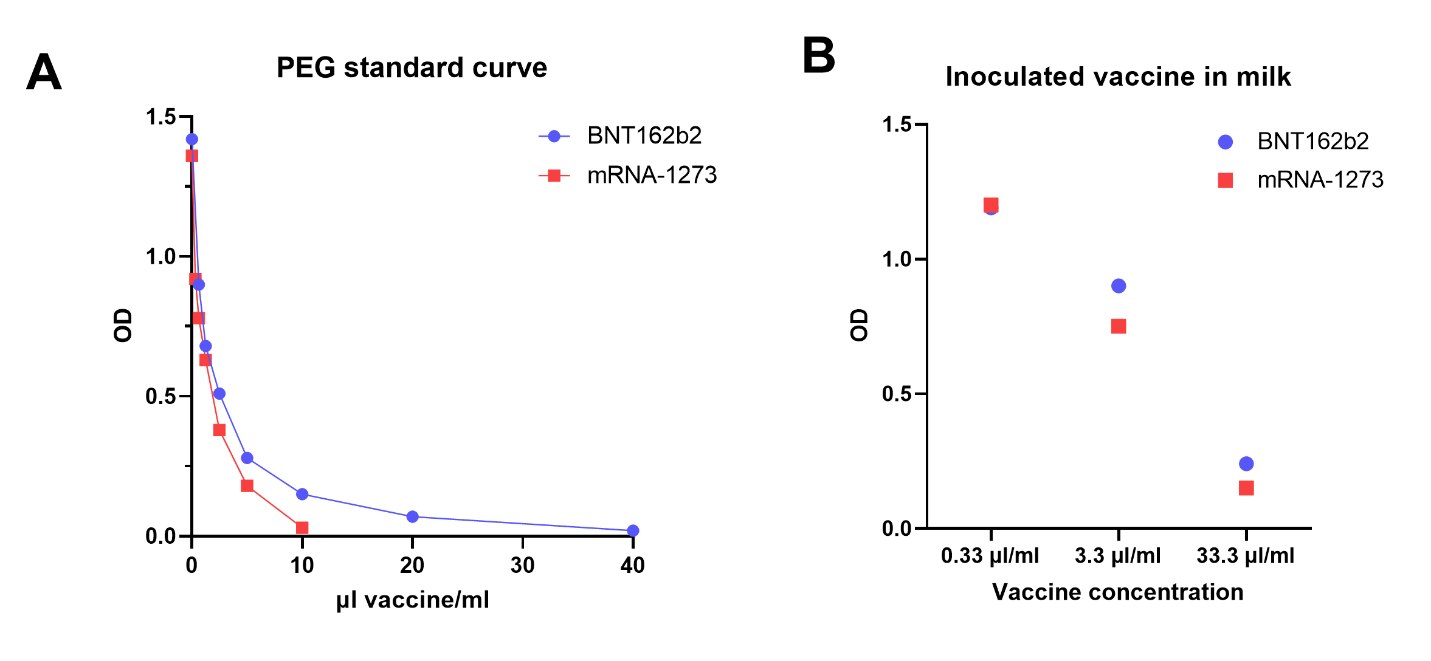


**Figure S1: Calibration of PEGylated protein assay.** A) different vaccine concentrations were used to generate a standard curve of OD (Y axis) vs. vaccine concentration (X axis). Vaccine concentrations in each sample were interpolated based on Sigmoidal, four-parameter logistic (4PL) curve. B) mRNA-1273 and BNT-162b2 vaccines were inoculated separately from pre-vaccine milk samples and were used to ensure the assay’s sensitivity to detect the vaccine PEG components in milk samples. Of note, PEGylated proteins concentration is higher in mRNA-1273 compared to BNT-162b2 which also stand in line with mRNA concentration in each vaccine (ready for administration vaccines were used).

**Table S1. Participants testing positive for SARS-CoV-2 during the study period**

| **Participants testing positive for SARS-CoV-2** |  |  |  |
| --- | --- | --- | --- |
| **Participant ID:** | 1 | 2 | 3 |
| **Mother positive for SARS-CoV-2** | No | **Yes** | **Yes** |
| **Infant positive for SARS-CoV-2** | **Yes** | **Yes** | No |
| **Other people in the household diagnosed** | Yes | N/A | No |
| **Time of diagnosis** | 1 week after 2nd dose | N/A | 10 day before 1st dose |
| **Baby exclusively breastfed** | No | Yes | No |
| **Maternal blood IgG (RFU)** |  |  |  |
| On the day of 2nd dose | 244 | 5503 | N/A |
| 4 weeks after 2nd dose | 2558 | 5290 | N/A |
| **Infant blood IgG (RFU)** |  |  |  |
| 4 weeks after 2nd vaccine dose | N/A | 1928 | N/A |
| **Infant blood IgA (U/ml)** |  |  |  |
| 4 weeks after 2nd vaccine dose | N/A | 122 | N/A |
| **Milk anti-RBD IgG levels (U/ml)** |  |  |  |
| Pre-vaccine | N/A | 55 | 7.3 |
| On the day of 2nd dose | 246 | 2653 | 323 |
| 4 weeks after 2nd dose | 375 | 2834 | 250 |
| **Maternal blood positive for anti SARS-CoV2-N-protein antibodies** | N/A | Samples collected on day of 2nd dose and 5 weeks after 2nd dose were positive. | N/A |
| **Maternal injection site symptoms** |  |  |  |
| Reported after 1st vaccine dose: | Pain | Pain, Itching | None |
| Reported after 2nd vaccine dose: | Pain | None | Pain |
| **Maternal generalized symptoms** |  |  |  |
| Reported after 1st vaccine dose: | None | None | None |
| Reported after 2nd vaccine dose: | Fever, Chills, Muscle aches or body aches, Fatigue or tiredness | Fever, Chills, Fatigue or tiredness | Fatigue or tiredness |
| **Baby symptoms** |  |  |  |
| After 1st dose | None | None | None |
| after 2nd dose: | None | Less active. Feverish. | None |
| Exclusively breastfed | No | Yes | Yes |

**Table S2. Correlations between antibody levels and timing of samples in relation to childbirth and vaccine**

| Antibodies and sample types being correlated | N | Spearman correlations | |
| --- | --- | --- | --- |
|  |  | rho | p |
| Samples collected after 1st dose |  |  |  |
| IgG in maternal blood and … | 24 |  |  |
| Time from childbirth to collection |  | 0.38 | 0.07 |
| Time from 2nd dose to sample |  | -0.11 | 0.59 |
| IgG in breast milk and … | 35 |  |  |
| Time from childbirth to sample |  | -.05 | 0.75 |
| Time from 2nd dose to sample |  | -0.12 | 0.47 |
| IgA in breast milk and … | 38 |  |  |
| Time from childbirth to sample |  | -0.18 | 0.28 |
| Time from 2nd dose to sample |  | 0.12 | 0.45 |
| Samples collected after dose 2 |  |  |  |
| IgG in maternal blood and … | 32 |  |  |
| Time from childbirth to sample |  | 0.01 | 0.92 |
| Time from 2nd dose to sample |  | -0.34 | 0.05 |
| IgG in breast milk and … | 44 |  |  |
| Time from childbirth to sample |  | 0.04 | 0.75 |
| Time from 2nd dose to sample |  | 0.21 | 0.16 |
| IgA in breast milk and … | 43 |  |  |
| Time from childbirth to sample |  | **-0.35** | **0.02** |
| Time from 2nd dose to sample |  | -0.17 | 0.27 |

**Table S3. Antibody levels were not significantly correlated with maternal BMI**

| Correlation between maternal BMI and the following antibody levels: | n | Spearman correlations | | |
| --- | --- | --- | --- | --- |
|  |  | rho | p |  |
| After 1st dose |  |  |  |  |
| IgG in maternal blood | 24 | -.071 | .74 |  |
| IgG in milk | 35 | -.029 | .87 |  |
| IgA in milk | 38 | -.204 | .22 |  |
| 4-10 weeks after 2nd dose |  |  |  |  |
| IgG in maternal blood | 32 | .113 | .54 |  |
| IgG in milk | 44 | -.101 | .51 |  |
| IgA in milk | 43 | .115 | .46 |  |

**Table S4. Follow up maternal and infant blood samples characteristics and anti-SARS-CoV2 IgG levels**

| Participant # | Participant Cohort: | Infant sex | Infant age at sample date (months): | Infant age at 1st dose (months) | Time since 1st dose until infant sample collection (days) | Infant anti-SARS-CoV2 IgG antibodies (RFU): | Maternal anti-SARS-CoV2 IgG antibodies (RFU): | Vaccine type: |
| --- | --- | --- | --- | --- | --- | --- | --- | --- |
| 1 | Lactating | Female | 0-3 | 0-3 | 84 | 6 | 2768 | mRNA-1237 |
| 2 | Lactating | Male | 0-3 | 0-3 | 58 | 2 | 1791 | mRNA-1237 |
| 3 | Lactating | Female | 0-3 | 0-3 | 64 | 0 | 1638 | BNT162b2 |
| 4 | Lactating | Female | 3-6 | 0-3 | 67 | 4 | 5028 | mRNA-1237 |
| 5 | Lactating | Female | 3-6 | 0-3 | 61 | 6 | 3449 | mRNA-1237 |
| 6 | Lactating | Male | 6-9 | 3-6 | 81 | 7 | 3292 | mRNA-1237 |
| 7 | Lactating | Male | 9-12 | 6-9 | 103 | 2 | 1574 | mRNA-1237 |
| 8 | Lactating | Male | >12 | 9-12 | 62 | 3 | 3418 | BNT162b2 |

Note: negative values in infant age represent days before delivery. RFU>50 considered as positive value.
